# A Novel Prognostic Model of Hepatocellular Carcinoma per Two NAD+ Metabolic Synthesis-Associated Genes

**DOI:** 10.3390/ijms251910362

**Published:** 2024-09-26

**Authors:** Luo Dai, Shiliu Lu, Linfeng Mao, Mingbei Zhong, Gangping Feng, Songqing He, Guandou Yuan

**Affiliations:** 1 Division of Hepatobiliary Surgery, The First Affiliated Hospital of Guangxi Medical University, Nanning 530021, China; dl_dailuo@163.com (L.D.); shiliulu@126.com (S.L.); gxmumlf@163.com (L.M.); 18277640805@163.com (M.Z.); gangpingfeng@163.com (G.F.); 2 Key Laboratory of Early Prevention and Treatment for Regional High Frequency Tumor (Guangxi Medical University), Ministry of Education, Nanning 530021, China; 3 Guangxi Key Laboratory of Immunology and Metabolism for Liver Diseases, Nanning 530021, China

**Keywords:** hepatocellular carcinoma (HCC), prognostic model, NAD+, PARP2, SIRT6

## Abstract

Hepatocellular carcinoma (HCC) is a formidable challenge to global human health, while recent years have witnessed the important role of NAD+ in tumorigenesis and progression. However, the expression pattern and prognostic value of NAD+ in HCC still remain elusive. Gene expression files and corresponding clinical pathological files associated with HCC were obtained from the Cancer Genome Atlas (TCGA) database, and genes associated with NAD+ were retrieved from the GSEA and differentially analyzed in tumor and normal tissues. A consensus clustering analysis was conducted by breaking down TCGA patients into four distinct groups, while Kaplan–Meier curves were generated to investigate the disparity in clinical pathology and endurance between clusters. A prognostic model based on NAD+-associated genes was established and assessed by combining LASSO-Cox regression, uni- and multi-variate Cox regression, and ROC curve analyses. Investigations were conducted to determine the expression of distinct mRNAs and proteins in both HCC and non-tumor tissues. A novel two-gene signature including poly (ADP-Ribose) polymerase 2 (PARP2) and sirtuin 6 (SIRT6) was obtained through LASSO-Cox regression and was identified to have favorable prognostic performance in HCC patients from TCGA. Analyses of both single and multiple variables showed that the prognostic model was a distinct prognostic factor in the endurance of liver cancer patients in both the training and trial groups. The nomogram also exhibited clinical significance in the prognosis of HCC patients. Immunohistochemistry, qRT-PCR, and Western blotting revealed that HCC samples exhibited higher PARP2 and SIRT6 expression levels than those of normal controls. This study identified a robust prognostic model comprising two NAD+-associated genes using bioinformatic methods, which is accurate in predicting the survival outcome of HCC patients. This model might benefit the early diagnosis of HCC and further facilitate the management of individualized medical service and clinical decision-making.

## 1. Introduction

HCC is a prevalent malignancy that ranks third in global fatality among cancers [1]. According to WHO statistics, almost over nine hundred thousand people have been diagnosed with HCC [2]. It is a great challenge to cure the disease due to it being highly heterogeneous, readily resistant to chemoradiotherapy, hard to be diagnosed in early stages, progressing rapidly and aggressively, and lacking high-efficient target drugs. In this context, exploring a significant model in the early diagnosis, treatment, and prognosis of HCC is critically important for HCC patients’ survival.

**Figure 1:**
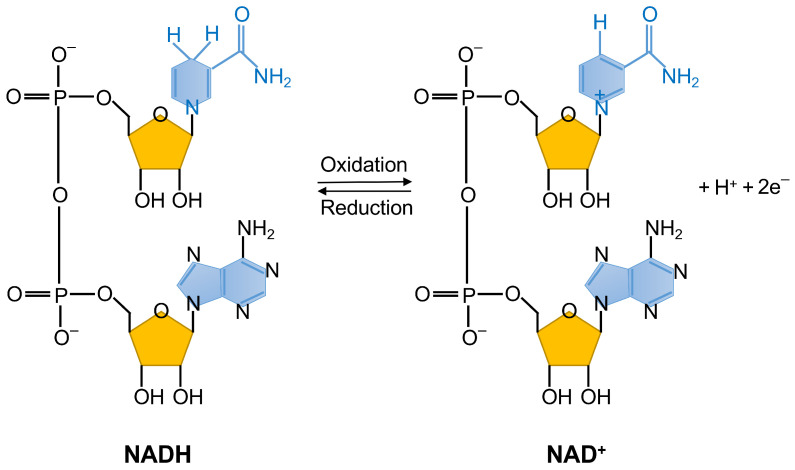
The structures of NAD+ and NADH.

NAD+ and NADH, the primary components of nicotinamide adenine dinucleotide (NAD), are fundamental coregulators in the organism [3] (Figure 1). NAD+ participates in a variety of biological activities, such as energy metabolism, DNA repair, cellular metabolism, and signal transduction. Initial research revealed that NAD+ levels can be a predictor of a range of illnesses, such as degenerative disorders of the central nervous system, cardiovascular illness, and tumors [4]. High levels of energy metabolism are always required to sustain the unlimited growth of tumor cells in tumorigenesis. A previous study reported that *CD38* could induce cell cycle arrest by significantly decreasing the NAD+ level in prostate cancer cells, which might be the key mechanism involved in the delay of disease progression [5]. In mammalian cells, the rate-limiting enzyme nicotinamide phosphoribosyltransferase (*NAMPT*) has a major effect on NAD biosynthesis through the salvage pathway [6]. In addition, *NAMPT* exhibits abnormal expression in multiple tumor tissues and is predictive for poor prognosis [7,8,9]. Meanwhile, *NAMPT* inhibitor *FK866* is capable of accelerating tumor cell death, concomitantly with reductions in *NAMPT* expression and the NAD+ level [10]. This study implies that *NAMPT* inhibitors might be viable options in the treatment of HCC. Collectively, NAD+-related genes might play a significant role in tumorigenesis and progression.

To obtain datasets about clinical information and patient gene expression based on studies, sequencing files associated with HCC were retrieved from TCGA databases. For the TCGA dataset, gene expression files were studied in 32 NAD+-associated protein-coding genes. Our bioinformatics-based study explored the key regulators of NAD+ biosynthesis, which could predict the prognosis of HCC patients. This study aims at providing new strategies and thoughts toward the treatment of HCC.

## 2. Results

### 2.1. NAD+-Associated Differentially Expressed Genes (DEGs)

**Figure 2:**
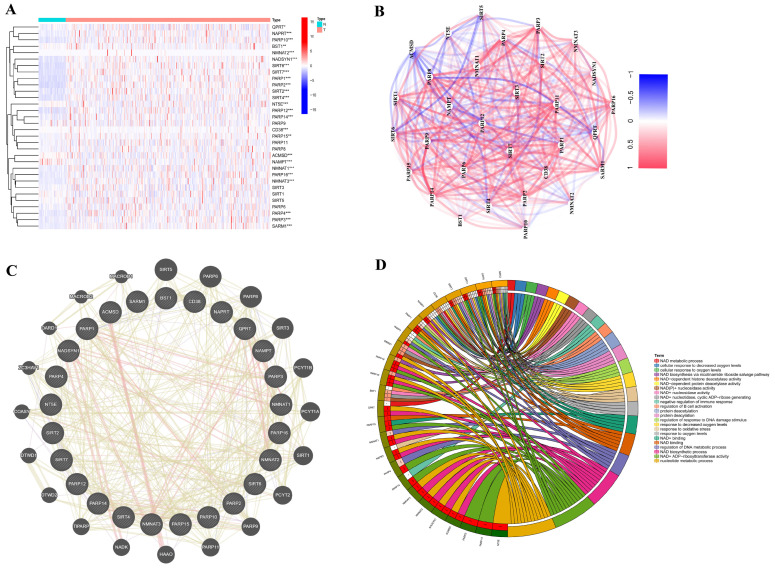
Expression of 32 NAD+-related genes in hepatocellular carcinoma and their correlation, as well as biological regulation of interacted proteins. (**A**) Heat map of expression difference in NAD+-related genes. (**B**) Heat map of correlation between DEGs. (**C**) PPI network map of DEGs. (**D**) DEGs involved in biological regulation of related proteins.

To explore NAD+-associated genes, expression files of 32 NAD+-associated protein-coding genes in the TCGA dataset (tumor, n = 374; normal, n = 50) were extracted and analyzed. We found that 25 genes exhibited differential expression between tumor and normal tissues, including *NTE5*, *QPRT*, *ACMSD*, *BST1*, *NADSYN1*, *NAMPT*, *NAPRT*, *NMNAT1*, *CD38*, *NMNAT2*, *NMNAT3*, *PARP1*, *PARP10*, *PARP12*, *PARP14*, *PARP15*, *PARP16*, *PARP2*, *PARP3*, *PARP4*, *SARM1*, *SIRT2*, *SIRT4*, *SIRT6*, and *SIRT7* (Figure 2A). We also found that these DEGs showed abundant correlation networks and protein–protein interaction (PPI) regulatory networks (Figure 2B,C). In addition, gene ontology (GO) enrichment analysis revealed that these 25 DEGs were mainly involved in NAD+ biosynthesis and metabolism (Figure 2D).

### 2.2. Consensus Clustering Analysis

**Figure 3:**
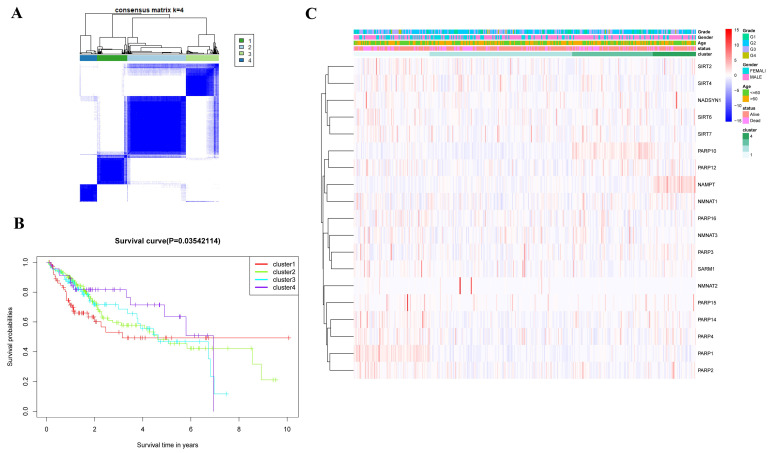
The consensus clustering analysis of the NAD+ regulators. (**A**) The most appropriate selection with clustering stability when k = 4 was used. (**B**) The overall survival rates of different clusters were different. (**C**) Heat maps of the relationship between different clusters and clinicopathology.

Next, we performed consensus clustering analysis in 374 HCC patients from the TCGA database, from clusters (K) from 2 to 9. When K = 4, the intergroup correlation was weak, while the intragroup correlation was strong (Figure 3A). A significant difference was demonstrated across the four clusters in terms of the survival rate as analyzed by the Kaplan–Meier (KM) method (Figure 3B). In contrast, our assay did show significant differences regarding tumor grade, patient age, and gender (Figure 3C).

### 2.3. Establishment and Validation of Prognostic Model

**Figure 4:**
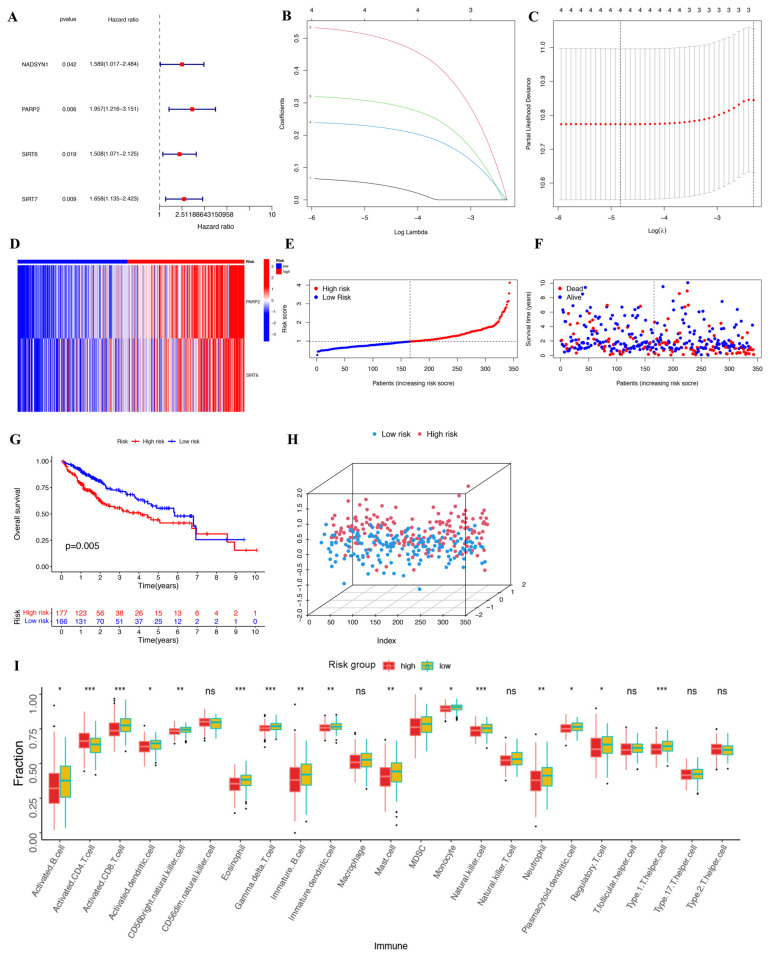
The risk model of the NAD+ regulatory factor was constructed based on TCGA hepatocellular carcinoma (HCC) data. (**A**) With *p* < 0.2 as the standard, 4 genes related to OS were screened by uni-variate regression analysis. (**B**) Target genes were screened as prognostic models by LASSO. (**C**) Cross-validation is intended to adjust parameter selection in LASSO regression. (**D**) A heat map of the relationship between the risk score and 2 genes. (**E**) HCC patient distribution based on the risk score. (**F**) HCC patient survival status (a dotted line is used to identify patients with high or low risk scores). (**G**) A Kaplan–Meier plot was used to compare OS between the high- and low-risk groups. (**H**) A PCA diagram based on the risk score. (**I**) The ssGSEA method was applied to the HCC samples to evaluate the distribution of 23 immune cell types. * *p* < 0.05,** *p* < 0.01, *** *p* < 0.001.

**Figure 5:**
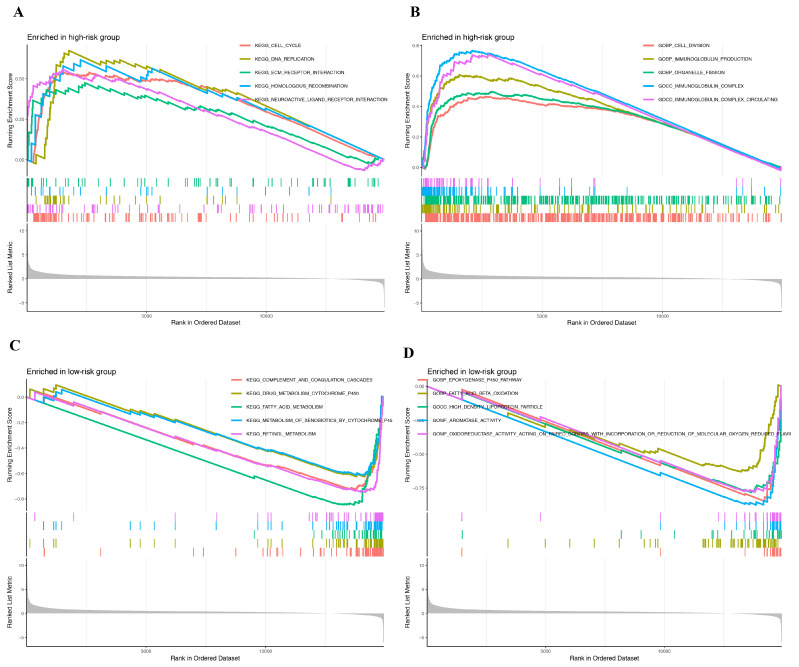
GESA analysis based on GO and KEGG. (**A**) The top 5 most significantly enriched KEGG pathways in the high-risk group. (**B**) The top 5 most significantly enriched GO terms in the high-risk group. (**C**) The top 5 most significantly enriched KEGG pathways in the low-risk group. (**D**) The top 5 most significantly enriched GO terms in the low-risk group.

**Figure 6:**
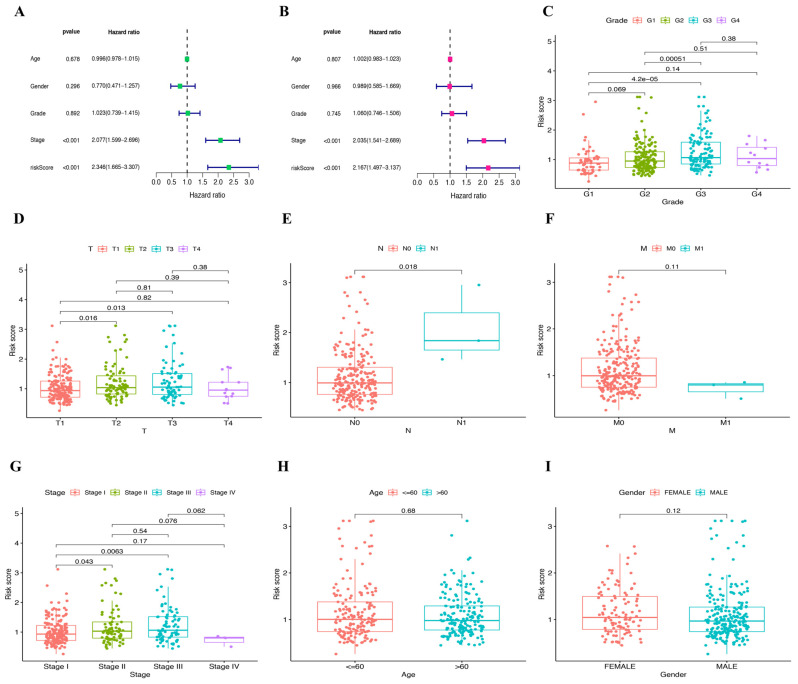
Uni-variate and multi-variate regression analyses were used to assess the prognostic value of risk scores in TCGA. (**A**,**B**) Uni-variate and multi-variate regression analyses in TCGA. (**C**–**I**) The correlation between the risk score and clinical features of patients from TCGA.

Next, uni-variate Cox regression analysis was performed, and four DEGs were identified to be prognostic for the survival (*p* < 0.05) of HCC patients, including *PARP2*, *SIRT6*, *SIRT7*, and *NADSYN1* (Figure 4A). In addition, Lasso-Cox regression was performed to combine the above genes (Figure 4B,C). A heat map of *PARP2* and *SIRT6* indicated that these two genes exhibited high risk scores and were further used to establish a prognostic model (Figure 4D). A risk score was correspondingly generated and divided TCGA cancer patients into high- and low-risk groups based on the median risk value (Figure 4E,F). Survival analysis revealed that the patients of the high-risk group survived for a shorter amount of time and were more prone to death than the patients of the low-risk group (Figure 4G), suggesting that higher risk scores were indicative of shorter survival times. Furthermore, principal component analysis (PCA) was performed and proved that the two groups of patients were well distinguished (Figure 4H). To further examine the relationship between risk score and immune cell infiltration, we performed ssGSEA analysis, and the data revealed the characteristics of high-risk and low-risk groups, and 23 immune cell types indicated that the risk score was correlated with immune response. In comparison to the high-risk group, a marked increase in the activation of B cells, CD8 T cells, and dendritic cells was observed in the low-risk group (Figure 4I). Based on the GSEA analysis of GO, Kyoto Encyclopedia of Genes and Genomes (KEGG), we found that compared to the low-risk group, at least five KEGG pathways were significantly enriched in the high-risk group, which include the cell cycle, DNA replication, ECM receptor interaction, Homologous recombination, and Neuroactive ligand receptor interaction (Figure 5A,C). The GSEA analysis of GO also showed that pathways such as Cell division, Immunoglobulin production, Organelle fission, Immunoglobulin complex, and circulating Immunoglobulin complex were enriched in the high-risk group but not in the low-risk group (Figure 5B,D). Moreover, uni-variate Cox regression analysis showed that the risk score was prognostic for poor survival in patients from TCGA (HR = 2.346, *p* < 0.001) (Figure 6A). Multi-variate Cox regression analysis demonstrated that the risk score was an independent risk factor for the survival outcome of patients from TCGA (HR = 2.167, *p* < 0.001) (Figure 6B). Finally, related graphs were generated to show the correlation between the risk score and clinical features of patients from TCGA-HCC (Figure 6C–I).

### 2.4. Nomogram Was Constructed to Predict Prognosis of HCC Patients

**Figure 7:**
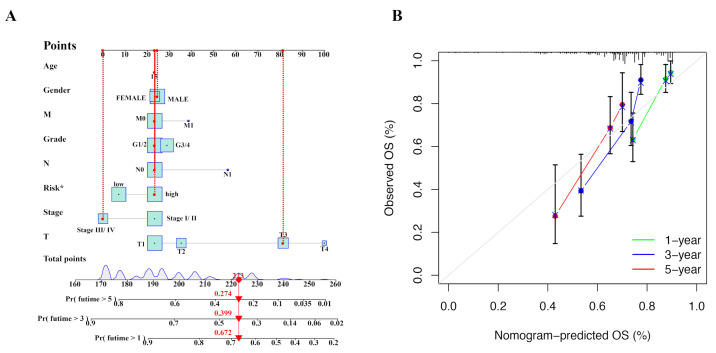
Nomogram for evaluating prognosis. (**A**) Nomogram was applied to assess prognosis of HCC patients in TCGA. (**B**) Calibration curve for predicting 1-, 3-, and 5-year prognosis of HCC patients.

The nomogram was constructed to predict the 1-, 3-, and 5-year prognosis of HCC patients using data from TCGA (Figure 7A). The diagonal line at 45 degrees signifies an ideal predictive model, and the calibration plot obtained suggests that the nomogram performed effectively (Figure 7B).

### 2.5. Verification of Clinical Tissue Samples

**Figure 8:**
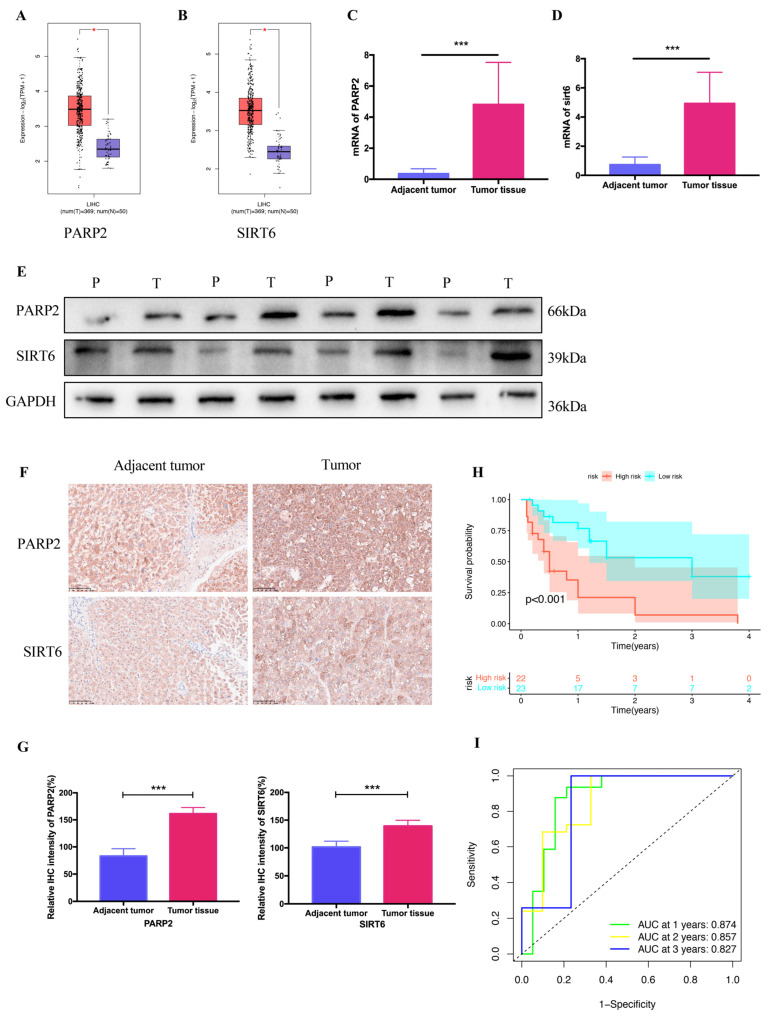
Verification of clinical tissue samples. (**A**,**B**) Expression of PARP2 and SIRT6 in HCC tissues and non-tumor tissues analyzed by GEPIA2.0. (**C**,**D**) RT-qPCR analysis of PARP2 and SIRT6 in HCC and non-tumor tissues. (**E**) Protein level of PARP2 and SIRT6 in HCC and non-tumor tissues. (**F**) Characteristic pictures of PARP2 and SIRT6 in HCC and adjacent tumor. (**G**) Statistical diagram of F. (**H**) Survival analysis between patients in high-risk and low-risk group. (**I**) ROC curve was used to evaluate predictive effectiveness. All data are shown as mean ± standard deviation. *** *p* < 0.001.

Based on Gene Expression Profiling Interactive Analysis 2.0 (GEPIA2.0), *PARP2* and *SIRT6* were significantly upregulated in HCC tissues compared to those in non-tumor tissues (Figure 8A,B). Consistent with the results in GEPIA2.0, the mRNA expression of *PARP2* and *SIRT6* was significantly increased in HCC tissues compared to that in non-tumor tissues (Figure 8C,D). In addition, the protein levels of *PARP2* and *SIRT6* were also increased in HCC tissues (Figure 8E–G). An analysis of survival showed that the duration of life for those in the high-risk group was shorter than for those in the low-risk group (Figure 8H). The AUC values of the 1-, 2-, and 3-year ROC curves at 0.874, 0.857, and 0.827, respectively, indicate the model’s potential to be a prognostic agent (Figure 8I).

## 3. Discussion

Biochemically, NAD+ is known to be associated with tumorigenesis. However, the mechanism by which NAD+ regulates HCC remains unclear. In this study, we found that among the 32 NAD+-associated genes retrieved from GSEA, 25 genes show differential expression in tumor and normal tissues based on the data from TCGA. Consensus clustering analysis divided TCGA patients into four clusters with distinct differences in the survival rate. We also identified that two NAD+-associated genes, *PARP2* and *SIRT6*, are significantly prognostic for the survival (*p* < 0.05) of HCC patients via uni-variate and LASSO-Cox regression analyses. In HCC tissues, the mRNA and protein levels of *PARP2* and *SIRT6* were significantly higher than those of non-tumor tissues (*p* < 0.001). Thus, a prognostic model was established, as well as a nomogram combining the risk score, patient age, gender, and tumor grade, both of which merit reliability in clinical prognosis.

NAD+ biosynthesis is achieved mainly via the de novo synthesis pathway and salvage pathway [11]. With the collaboration of NAD+-consuming enzymes, such as sirtuins, *PARPs*, *CD38*, and *CD157*, NAD+ can be catalyzed into NaM and ADP-Ribose. In recent years, tumor research has become a major area of focus due to the abnormal production and utilization of NAD+. Recent studies reported that NAD+ has dual effects on tumorigenesis. Low levels of NAD+ trigger the reactive oxygen species (ROS)-dependent degradation of 15-hydroxyprostaglandin dehydrogenase, an identified tumor suppressor enzyme, thereby advancing the cellular epithelial–mesenchymal transition (EMT), which is considered the driver of tumor initiation and metastasis [12]. On the other hand, high levels of NAD+ potentiate glycolysis to increase cancer cell proliferation and improve survival, demonstrating its potential role in tumorigenesis as a risk factor [13]. Primary HCC is a common tumor worldwide and a common cause of cancer death [14]. Solute carrier family 25 member 20 (*SLC25A51*) is a mammalian mitochondrial NAD+ transporter. Research showed that glucose metabolism reprogramming including oxidative phosphorylation and glycolysis contribute to growth and metastasis by *SLC25A51* in HCC cells [15]. Mitochondrial Ca^2+^ uniporter (MCU) may impede the NAD+/*SIRT3*/*SOD2* pathway, thereby stimulating ROS production in HCC cells [16]. It is difficult to cure primary HCC due to difficulties in early diagnosis and treatment. Therefore, developing a novel prognostic model is conducive to formulating an individualized and precise treatment strategy.

*PARP2* is a member of the poly (ADP-Ribose) polymerase family that was first discovered in embryonic fibroblasts of *PARP1*-deficient mice [17]. It has promotive effects on gastric cancer cell migration and spread, which can be reversed by upstream miR-128 [18]. The lncPTTG3P/miR-383/*PARP2* axis was reported to play a part in cancer cell invasion and migration in HCC [19]. PARPi alone, including *PARP1* and *PARP2*, or combined with radiotherapy contributed to HCC treatment, particularly for HBV-associated HCC [20]. Studies suggested that *PARP2* is a proto-oncogene under the regulation of multiple upstream non-coding RNAs. Nuclear members of the sirtuin family of NAD+-dependent deacetylases, sirtuin 6/7 (*SIRT6/7*), are known as sirtuin 6/7. In the heterochromatin region, *SIRT6* is found to possess deacetylase and ADP-ribosyl transferase activities. The role of *SIRT6* in tumorigenesis and development remains controversial, although it can act as an oncogene in breast cancer [21,22], prostate cancer [23,24], HCC [25], and diffuse large B-cell lymphoma by inducing cancer cell proliferation, metastasis, and chemoresistance [26]. In contrast, *SIRT6* can suppress aerobic glycolysis in tumor cells to inhibit cell growth and the cancerization of embryonic fibroblasts [27]. By means of the *JAK2*/*STAT3* signaling pathway, *SIRT6* can inhibit tumor cell growth by inducing apoptosis and diminishing oxidative stress [28,29,30]. Some studies point to the complexity of *SIRT6*’s biological role in tumorigenesis. Here, we found that *SIRT6* being differentially expressed in HCC tissues suggests that it might be associated with a poor prognosis in HCC patients.

Nevertheless, there are still some limitations. For example, the quality and quantity of publicly available samples may affect the authenticity of this study, and the expression of the model genes requires to be further validated by experiments. In the present, there exist a variety of traditional treatments, including radiofrequency ablation, transcatheter arterial chemoembolization, selective internal radiation therapy, etc. Therefore, the prognostic model in this study only provides a new idea for clinical treatment. If it needs to be applied in clinical treatment, a larger number of clinical samples need to be included for verification. Additionally, the results are not compared to established predictors, which is something we need to improve in future work.

## 4. Materials and Methods

### 4.1. Tissue Samples

This study was approved by the Clinical Research Ethics Committee of the First Affiliated Hospital of Guangxi Medical University. This study used surgically removed HCC tissue (n = 32) and matched adjacent tumor tissue (n = 32) that were stored in liquid nitrogen or fixed in 10% formalin solution, which were then dehydrated and embedded in paraffin for preservation.

### 4.2. Data Sources

Sequencing data of HCC and paired clinical data were retrieved from TCGA “https://www.cancer.gov/tcga (accessed on 28 May 2024)”. All data are publicly available, and there are no competing interests or copyright issues.

### 4.3. Screening of DEGs Associated with NAD+

NAD+-associated genes were retrieved by GSEA. The package “limma” was used to screen out the DEGs between cancer tissues and normal tissues from TCGA, and the package “igraph” was adopted to visualize associations between the DEGs in a heat map. The GeneMANIA online tool was applied to construct a PPI network [31]. The GO plot package was utilized to look for significant GO entries of the DEGs.

### 4.4. Establishment and Validation of Prognostic Model

A uni-variate Cox regression model was built to look for prognostic genes for survival in TCGA patients using the R package “survival” (*p* < 0.2). A LASSO-Cox regression model was adopted to establish a prognostic model based on the genes. A model-based risk score was correspondingly generated. Grouping each patient in TCGA into high- and low-risk groups was conducted by scoring them on the median value. The two groups’ KM curves were plotted. The ssGSEA method was applied to the TCGA-HCC samples to evaluate the distribution of 23 immune cell types. A correlation between the risk score and clinical characteristics, including patient age, gender, and tumor grade, was visualized by column charts. Uni- and multi-variate Cox regression models were constructed to assess whether the risk score is an independent risk factor for the prognosis of HCC patients. A nomogram was plotted using the package “rms”, while the calibration and ROC curves were used to determine the dependability of the nomogram.

### 4.5. Immunohistochemistry (IHC)

After two hours in an oven at 60 °C, the sections fixed with paraffin were dewaxed in xylene for 10 min. Different concentrations of alcohol (100%, 95%, 80%, 75%, 50%) were used for hydration treatment, and the sections were placed in a pressure cooker for 15 min following EDTA antigen repair solution (C1038, Solarbio, Beijing, China). Endogenous catalase was removed and washed with PBS for 5 min 3 times, following the incubation with primary antibodies (PARP2, Proteintech, Chicago, IL, USA, 1:200; SIRT6, Proteintech, Chicago, IL, USA, 1:200) overnight in a wet box at 4 °C. On the second day, the slides were incubated with secondary antibody for 45 min at 37 °C, following DAB (ZLI-9018, Zsbio, Beijing, China) staining and nucleus counterstaining with hematoxylin (C0107, Beyotime, Shanghai, China).

### 4.6. Reverse Transcription Quantitative Polymerase Chain Reaction (RT-qPCR)

Total RNA was isolated from HCC and paracancer tissues for cDNA generation. Real-time PCR was then performed (PARP2: forward primer: TAC ACC AGG ATT CCG CAT GAC, reverse primer: TGG GTG TTC TGG GCT TTG TAG; SIRT6: forward primer: ATG TGC CAA GTG TAA GAC GCA, reverse primer: CTT GCC TTA GCC ACG GTG C). All procedures were carried out in accordance with the manufacturer’s instructions. The amplified thermal cycle parameters were as follows: 95 °C for 3 min, followed by 39 cycles of 95 °C for 10 s, 60 °C for 30 s, then 95 °C for 5 s, melt curve 65 °C to 95 °C, and increment 0.5 °C.

### 4.7. Western Blotting

Proteins from liver cancer tissues and paracancer tissues were collected in protein lysate buffer and subjected to Western blot assay with antibodies against PARP2 (Proteintech, Chicago, IL, USA, 1:2000) and SIRT6 (Proteintech, Chicago, IL, USA, 1:2000). The imprinting was visualized with ECL detection (SQ201, YAMEI, Shanghai, China), and an imaging system (SYNGENE, San Diego, CA, USA) was used to capture the chemiluminescence signal.

## 5. Conclusions

In conclusion, this is the first study that generates a prognostic model for HCC based on the genes associated with NAD+ synthesis and metabolism. The model contains two NAD+-related genes and shows potential prognostic capability in HCC. Thus, this study enriches the molecular mechanisms involved in the regulation of HCC and provides potential diagnostic biomarkers for HCC. Nevertheless, future studies regarding the biological functions of NAD+-related genes in HCC cells are needed, despite the limitation in the authenticity of the quality and quantity of publicly available samples.

## Data Availability

The data presented in this study are available on request from the corresponding author.

## Abbreviations

DEGs	Differentially expressed genes
EMT	Epithelial–mesenchymal transition
GEPIA2.0	Gene expression profiling interactive analysis 2.0
GO	Gene ontology
HCC	Hepatocellular carcinoma
IHC	Immunohistochemistry
KEGG	Kyoto encyclopedia of genes and genomes
KM	Kaplan–Meier
MCU	Mitochondrial Ca^2+^ uniporter
NAD	Nicotinamide adenine dinucleotide
NAMPT	Nicotinamide phosphoribosyltransferase
PARP2	Poly (ADP-Ribose) polymerase 2
PCA	Principal component analysis
PPI	Protein–protein interaction
ROS	Reactive oxygen species
RT-qPCR	Reverse transcription quantitative polymerase chain reaction
SIRT6	Sirtuin 6
SIRT6/7	Sirtuin 6/7
SLC25A51	Solute carrier family 25 member 20
TCGA	The cancer genome atlas
